# Supplementary material for: Facilitators and barriers to the implementation of a Mobile Health Wallet for pregnancy-related health care: A qualitative study of stakeholders’ perceptions in Madagascar
Source: PLoS One. 2020 Jan 31;15(1):e0228017. doi: 10.1371/journal.pone.0228017 (PMC6993972; doi:10.1371/journal.pone.0228017)
Supplement: S1 Appendix — (PDF) [file pone.0228017.s001.pdf]

IN-DEPTH-INTERVIEW GUIDE

**WP1. Questions (health officials):**
What is your position and your main responsibilities and main tasks within this health institution?
Financing health care is one of the major obstacles in access to appropriate care. Where do you see the main issues regarding financing health care for pregnant women in Madagascar?
Issues with financing health care is that women often don't have accessible savings and costs are high. A savings solution within our program is related to mobile money like Telma mVola, Airtel Money or Orange Money, we will talk about this later.<br>In general, do you have any experience with mobile payments within the healthcare sector you are supervising? I.e. payment of salaries, transfer of funds from CSB level to district level, mobile payment by patients etc.?<br>Where do you see the potential strengths and weaknesses of mobile payments within healthcare financing?
Now, we want to explain more about our new program and we are very interested in hearing from you about your impression, thoughts and concerns. Please tell me if the description is read too quick or if you have any problems to understand me. (read aloud text A). Probe for questions and understanding.<br>What are your first impressions of the described program?<br>What do you think especially about the mobile health wallet?
With mTomady, all payments would come through Mobile Money. Would you see this as beneficial or would there be any problems related to that?
Where do you see crucial obstacles from your perspective? (probe for technical issues, regulations, cultural aspects)
As said, this program should serve as a savings tool for pregnant women for healthcare services. Furthermore, finance transfer in CSBs shall get more transparent. Quality of care shall be enhanced by providing equipment, training, mobile ultrasound and free ambulance service for emergencies.<br>a. How do you think should an information campaign for this program look like to achieve that women will sign up for this program?<br>b. How do you think does this program needs to be presented to CSB staff to motivate them in participating in this program?
We are trying to make the most effective and informative messaging as possible in order to make it easier for people to access mTomady. Please think of a health campaign about another project or initiative that, in your opinion or in the opinion of others in the community, was done well. Think broadly.<br>a. Could you please tell us about it?<br>b. What makes you feel that this campaign was well done? What did you as a healthcare official like about the message? What made it effective?

Now, please think of an initiative around reducing maternal and newborn mortality that, in your opinion or in that of others, was not done well.<br>a.What makes you feel that this campaign was not done well?<br>b.What did people in the community not like about the message or the method?
What would be the most essential in order to have this project successful? What would you recommend to consider before implementing a program like mTomady?<br>And finally, is there anything that I didn't ask you that I should have asked you?<br>Is there anything else that you would like to add?
**WP2. Questions (phone network provider):**
What is your position and your main responsibilities and main tasks within your company?
Not being able to pay for healthcare is one of the major obstacles to healthcare access. Could you think about any reason why there is currently no broad mHealth approach enhancing healthcare access in Madagascar?
As I mentioned earlier the main focus of our discussion is a mHealth tool. Please think of any other mHealth tools related to Mobile Money in Madagascar. Have there been so far any developments? If yes, where do you see their strengths and weaknesses?
Now, we want to explain more about our new program and we are very interested in hearing from you about your impression, thoughts and concerns. Please tell me if the description is read too quick or if you have any problems to understand me (read aloud text A).<br>What are your first impressions of the described mobile health wallet?
Where do you see crucial obstacles from your perspective?<br>a. Concerning the implementation?<br>b. Concerning any regulations?<br>c. Concerning the marketing?<br>d. Concerning the cultural aspects?
What do you think would be the most essential in order to have this project successful?
Within your profession you deal with mobile money and payment partners. May we ask you to think of any mobile money partner with which one the cooperation went especially well.<br>a. What was essential to maximize the added value for both parties?<br>b. Have there been any obstacles within the partnership?
Has there been any campaign of a partner that you think went especially well?
In general, are there any advices you would like to give us on the basis of your experience?

And finally, is there anything that I didn't ask you that I should have asked you? Is there anything else that you would like to add?
**Questions (health care providers):**
What is your position and your main responsibilities and main tasks within this healthcare institution?
You are an expert on pregnancy and maternal health. Please think of the way how care for pregnant women is provided at your hospital. For you as a health care professional, are there any obstacles that make it difficult to care adequately for pregnant women? This could be the lack of equipment or lack of staff. Please think broadly. Do you have something in mind? (pause for confirmation). Please share your opinion with us.
Now, if you consider the obstacles, which, according to your experiences as a health professional, deter pregnant women from seeking care in CSBs or hospitals. What obstacles come to your mind? Please share with us your ideas. *(probe for distance, financial obstacles, lack of information, social barriers)*
Let's talk more about financial hurdles. What actions do you think need to be taken to help pregnant women to cope with the costs of care or to reduce their fears of high costs during childbirth or in case of cesarean section?
We are interested in your opinions and experiences with mobile money (Airtel Money, Orange Money or Telma Mvola). Imagine pregnant women would pay for treatment at your hospital / CSB using their mobile phone. Do you see this to be a good thing or a bad thing for you / the institution? Please explain your answer.
A facilitator reads aloud a simple description of the project activities (see text on the last page). Please tell me in your own words what you think about this description. There are no right or wrong words; I am looking to learn from your impressions and thoughts. All thoughts are welcome.<br>a. PROBES- What do you see as the main message from this description?<br>b. PROBES- Is there anything you didn't understand about the description?<br>c. PROBES- Is there anything you like about mTomady?<br>d. PROBES- Is there anything you don't like about mTomady?<br>e. PROBES- Do you think mTomady will be beneficial for your work at the CSB?<br>f. PROBES- Do you think mTomady will be beneficial for pregnant women?
We are trying to make mTomady most effective and helpful for you and pregnant women in order to make it easier for people to access good quality care during pregnancy and delivery. Can you think of anything else that we should consider to change/add for this project? Please explain.

We're now going to be talking about other campaigns to reduce maternal and newborn mortality. Think of a health campaign around pregnancy that, in your opinion or in the opinion of others in the community, was done well. It could be something that you have experienced yourself in your CSB/hospital, heard on the radio, read on a poster, learned about from a friend or during a community event. Think broadly. Do you have one in mind? (interviewer pauses for confirmation) Good. Now please tell me about that. What makes you feel that this campaign was well done? What did you as a healthcare professional like about the message? What made it effective?
Now, think of an initiative around reducing maternal and newborn mortality that, in your opinion or in that of others, was not done well. What makes you feel that this campaign was not done well? What did people in the community not like about the message or the method?
Are there other things you would like to say before we wind up?
**Questions (community health workers and community leaders):**
What is your position and your main responsibilities and main tasks within this health institution?
Financing medical health care is for pregnant women in Madagascar one of the major obstacles in access to appropriate care. Where do you see the main issues?
Do you think financial straits in this concern are a topic that men and women in Madagascar feel comforta-ble discussing with their family, friends and/or their partners? Why or why not?
Especially when unexpected complications during pregnancy and/or delivery occur a lot women struggle to raise the funds needed to cover the necessary interventions spontaneously. Which obstacles does women have to overcome to save enough money in advance?
Which possibilities of saving for these events do you know?
A facilitator reads aloud a simple description of the project activities (see text on the last page). Please tell me in your own words what you think about this description. There are no right or wrong words; I am look-ing to learn from your impressions and thoughts. All thoughts are welcome.<br>What are your first impressions of the described mobile health wallet?
Is there anything you don't like about this. Please tell me more about that.
Where do you see crucial obstacles from your perspective?<br>a. Concerning the implementation<br>b. Concerning any regulations<br>c. Concerning the marketing<br>d. Concerning the cultural aspects
What would be the most essential in order to have this project successful?
How would you advice our agents to proceed to reach as many pregnant women as possible?

Would you like to support your agents within their search for pregnant women?
What would you suggest is the most effective way of communication to launch the mobile health wallet?
Are there some things that you can imagine that will make it hard to convince women to use the mobile health wallet?
Can you think of anything else that we should consider in order to make it possible?
And finally, is there anything that I didn’t ask you that I should have asked you?<br>Is there anything else that you would like to add?

Text A. Project activities as presented to health officials & phone network provider representatives:
In Madagascar, maternal and neonatal mortality remain high despite efforts to reach the Millennium development goals. However, many complications and deaths during pregnancy and childbirth are due to preventable causes. As national surveys show, high costs of consultations and drugs are the main reasons why pregnant women do not participate in ANC or do not deliver at a health center.
In the last few years, the use of mobile phones has become widespread in Africa and in the footsteps of this revolution has followed mobile money. Mobile money allows users to send and receive money without the need for a bank account using only their mobile phone. The phone number serves as the account.
Our project aims at introducing a tool for financial inclusion in healthcare focusing on pregnant women. It will include 20.000 pregnant women in Antananarivo and will be done in cooperation with the Ministry of Health. The so-called mTomady mobile health wallet (MHW, portemonnaie mobile) allows users to save, send, receive, and pay money for pregnancy-related healthcare expenses. It makes use of mobile money, but with the crucial difference that money stored in the MHW can only be used to pay for healthcare services at collaborating providers. This makes saving for healthcare safer and increases transparency and accountability among providers. mTomady will also serve as a platform for governments, NGOs and donors to send money directly to patients making sure that money can only be used for healthcare.

Furthermore, the program focus also on quality of care in partner facilities, i.e. by providing training to healthcare staff, equipment, enhancing drug availability, offering mobile ultrasound and running an ambulance service for pregnancy related emergencies.

All in all, the program shall enable women to financially prepare for delivery, to increase quality of care during pregnancy and to enhance transparency of costs in CSB.

Text B. Project activities (ideal healthcare provider perspective):
“I’m a midwive at the CSB and I love caring for women in my community. Currently we take care for several hundred pregnant women. Everyday, we work hard to make sure that they have a safe pregnancy and that the day of delivery is a day of joy and not sorrow. This sometimes gets overwhelming. But with mTomady everthing is easier now.
When Nirina came to her first visit to my CSB she was scared about what the pregnancy meant for her financially. She did not know how to save for her care during pregnancy, not to mention delivery. But then she signed up to mTomady on her phone and I got my mTomady tablet. When she now comes to see me I can check whether the treatment she is coming for will be covered by mTomady. The only thing I have to do is typing in her phone number. I can be sure that her bills will be payed directly through mTomady, no cash involved. The best thing is all her ANCs will be covered by mTomady, including fees for her consultation and all drugs. So, I can focus on what I love to do, caring for woman like Nirina and help them to have a safe pregnancy. After Nirina’s visits I use my mTomady tablet

to type in information about her visit. This helps me keeping all information in one place and allows mTomady to send the reimbursement to the CSB. I could even get a bonus on my personal mobile phone for typing in the data. When Nirina was in her 20th week of pregnancy she had her first ultrasound. Yes, thanks to mTomady we can now offer free ultrasound for all mTomady patients right in our CSB. At ANC days once a month an mTomady team comes over to us with a machine and a generator and we get the data.
This way I was able to tell Nirina that for her delivery she has to go to a hospital and get a c-section because her placenta was in the wrong place.
Until the day of delivery Nirina still came to see me and I had the peace of mind that if there would be an emergency with her pregnancy I could always call the free mTomady ambulance service to bring her to the hospital immediately.
In the past, saving for a c-section has been very difficult for my patients. But with mTomady they now have a tool that helps them to save specifically for healthcare and earn bonuses when they do so. It makes their life and my life much easier because I know when they have mTomady it becomes much easier for them to afford the care they really need and I am able to deliver the high quality care I would like to give. mTomady also helps me even more to do so. Last week one of my Blood pressure cuffs broke. We had it for the last 10 years. Through mTomady I was able to apply for a free replacement, with a click of a button on my mTomady tablet.
I trust mTomady because it works with strong partners and everyone will give their best to make it a success: The Ministry of Health, Telma, Orange, Airtel, and Doctors for Madagascar – all join hands for mTomady. mTomady makes my live easier and offers me great benefits to increase my CSB's income and the quality of care."
